# Acute Cytotoxic Cerebellar Edema Subsequent to Fentanyl Patch Intoxication in an Infant

**DOI:** 10.1155/2021/9449565

**Published:** 2021-09-07

**Authors:** Lindsey N. Haut, Rupa Radhakrishnan, Riad Lutfi, Louise W. Kao, Laurie L. Ackerman

**Affiliations:** ^1^Division of Pediatric Emergency Medicine, Department of Emergency Medicine, Indiana University School of Medicine, Riley Hospital for Children, Indianapolis, IN, USA; ^2^Department of Radiology, Indiana University School of Medicine, Riley Hospital for Children, Indianapolis, IN, USA; ^3^Division of Pediatric Critical Care Medicine, Department of Pediatrics, Indiana University School of Medicine, Riley Hospital for Children, Indianapolis, IN, USA; ^4^Department of Toxicology, Indiana University School of Medicine, Riley Hospital for Children, Indianapolis, IN, USA; ^5^Department of Neurosurgery, Indiana University School of Medicine, Riley Hospital for Children, Indianapolis, IN, USA

## Abstract

The opioid epidemic continues to have devastating consequences for children and families across the United States with rising prevalence of opioid use and abuse. Given the ease of access to these medications, accidental ingestion and overdose by children are becoming increasingly more common. The recognition of opioid-induced neurotoxicity and the associated life-threatening complication of acute cerebellar cytotoxic edema are crucial, as are the high morbidity and mortality without timely intervention. We discuss an infant with acute cytotoxic cerebellar edema following mucosal exposure to a transdermal fentanyl patch.

## 1. Introduction

The United States (US) is experiencing unprecedented rates of drug overdose-related deaths. The mortality toll in the US attributable to opioid medications has doubled in last two decades with more than 130 people dying daily following an opioid overdose [1]. The misuse of opioids, including prescription pain relievers and synthetic opioids, is a serious national crisis that affects adults and children. The number of opioid-related hospitalizations has doubled in the last twenty years with the largest percentage increase found in children under 5 years old, and the number of children requiring admission to pediatric intensive care for opioid ingestions has doubled in the last decade [2, 3]. The increasing number of adult drug prescriptions is strongly associated with rising pediatric exposures and poisonings; young children are at the greatest risk for exposure, with substantial health care use and morbidity specifically associated with opioid ingestions [4].

Opioid-induced neurotoxicity in adults can present with a wide spectrum of symptoms, including confusion, hallucinations, delirium, and seizures. In contrast, the neurological effects of opioid intoxication in children are poorly understood. Rapid identification of these children may present a diagnostic dilemma, especially if a history of opioid ingestion is not available.

Malignant cerebellar edema secondary to opioid intoxication is rarely reported in children. Without a high clinical suspicion, this imaging presentation may be attributed to other vascular, infectious, or postinfectious etiologies which are more common in children [5, 6]. Here, we describe a case of an infant with malignant cerebellar edema due to an inadvertent exposure to a transdermal fentanyl patch that required a lifesaving decompression craniectomy. We present this case to heighten awareness, especially in the acute pediatric clinical team.

## 2. Patient Presentation

**Figure 1:**
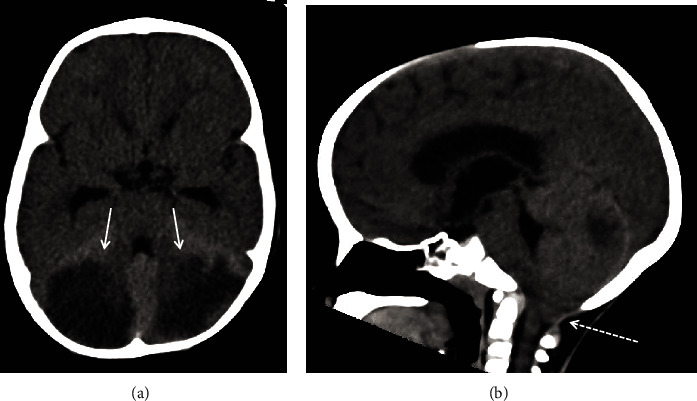
Axial (a) and midsagittal (b) noncontrast head CT at presentation shows bilateral cerebellar hemisphere hypoattenuation (low density) with swelling (arrows). There is inferior herniation of the cerebellar tonsils with crowding at the foramen magnum (dashed arrow).

The patient was a 9-month-old male with a past medical history significant for in utero drug exposure and mild oral aversion, who had otherwise been reportedly well. The day prior to his initial presentation, he had been swimming with his family and was put down for a nap at 1600. Family checked on him at 2100 hours due to not waking up for dinner but found him sleeping and attributed his increased fatigue to his increased activity throughout the day. At 0700 hours the next day, family was still unable to wake the child. He was taken to the nearest emergency department, where his initial vitals were significant for hypoxemia, with oxygen saturation of 77% on room air and mild hypothermia with axillary temperature of 36.2°C. His physical exam was notable for lethargy and decreased level of arousal without external physical exam findings concerning for apparent injury or infection. Labs were significant for hypoglycemia (30 mg/dL), but his mental status did not improve with glucose supplementation. A head CT was performed, and he was found to have bilateral cerebellar hemisphere hypoattenuation with acute swelling on computed tomography (Figure 1). The presumed diagnosis at the time was acute stroke of unknown etiology, with the possibility of prolonged hypoglycemia and subsequent hypoxia contributing. He was flown to the closest pediatric tertiary care center and admitted to the Pediatric Intensive Care Unit (PICU) for close neurological monitoring and further evaluation.

## 3. Initial Diagnosis and Outcome

**Figure 2:**
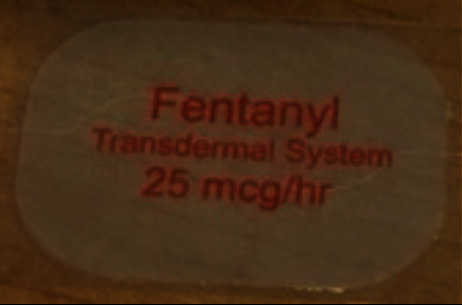
Example of fentanyl patch removed from patient's palate (provided by the caregiver).

On arrival at the PICU, he was found to have pinpoint pupils bilaterally without reactivity and a downward gaze, with withdrawal to painful stimulus. Due to concerns for the ability to protect his airway on arrival at the PICU, rapid sequence induction and intubation (RSI) was prepared, and on visualization of the patient's posterior oropharynx, a fentanyl patch was found adhered to his soft palate (Figure 2). The patch was easily removed from the mucosal surface. Intravenous naloxone was administered, and the patient immediately became more awake with conjugate pupils. However, his clinical improvement was transient, and RSI was completed to stabilize his airway and control ventilation.

Additional history revealed the patient's caretaker used fentanyl patches for chronic pain. The caretaker had replaced their patch prior to swimming the day before presentation and realized she was no longer wearing the patch shortly after arrival at the PICU. The exact length of time the patch was adherent to the patient's mucosal surface or the amount of medication remaining in the patch was unknown.

Toxicology was consulted, but there is little information available in regard to the pharmacokinetics of mucosal absorption of fentanyl. While case reports of pediatric patients after opioid pill ingestion are available, there were no references for fentanyl patch mucosal absorption. Serum toxicology testing was sent, with a serum fentanyl level from the day of admission returning elevated at 12 ng/mL (therapeutic level 1-4 ng/mL). Additional toxicology testing was negative (APAP, ASA, EtOH, amphetamine, cocaine, THC, opiate, PCP, and barbiturates). Standard urine drug screening for opioids is negative with fentanyl exposure due to its structural dissimilarity to morphine.

**Figure 3:**
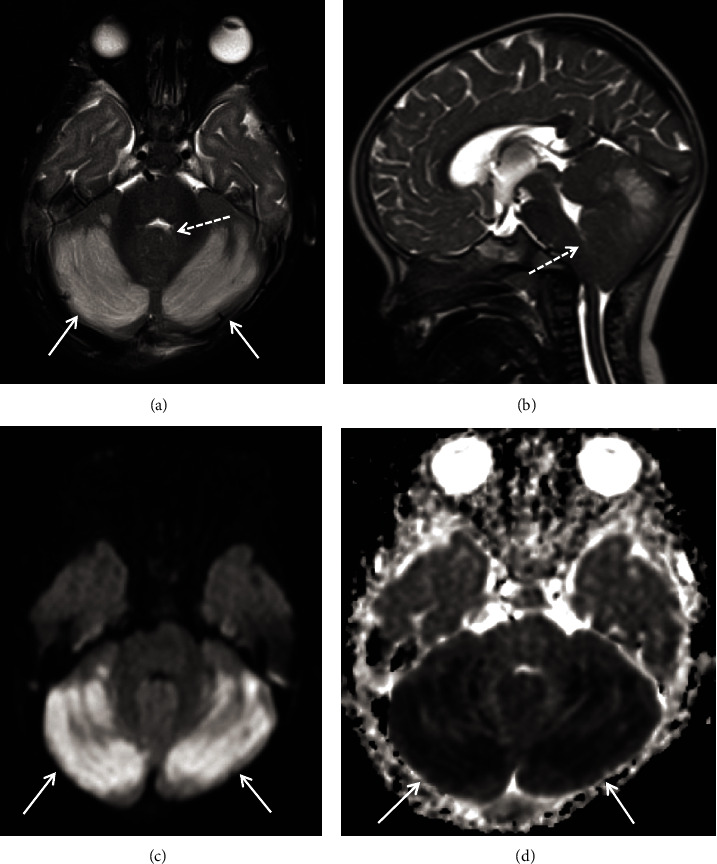
MRI at the time of presentation. (a) Axial T2-weighted image shows bilateral cerebellar edema (arrows) with effacement of the inferior fourth ventricle (dashed arrow). (b) Midsagittal T2 weighted image shows posterior fossa crowding with effacement of the inferior fourth ventricle (dashed arrow). (c) Diffusion imaging and (d) apparent diffusion coefficient (ADC) map show true restricted diffusion in the cerebellar hemispheres (arrows) in the region of T2 signal abnormality consistent with cytotoxic cerebellar injury.

**Figure 4:**
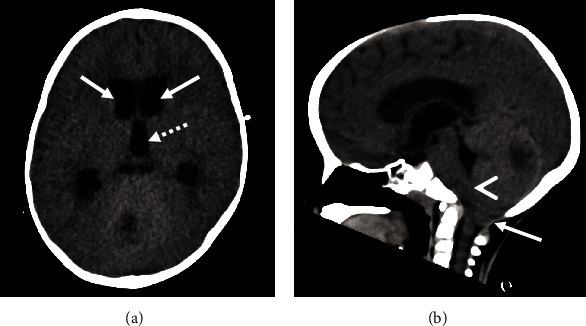
Noncontrast head CT images from hospital day 3 associated with neurological decline. (a) Axial CT image shows enlargement of the lateral (arrows) and third (dashed arrow) ventricles, and (b) midline sagittal CT image shows cerebellar swelling, effacement of the inferior fourth ventricle (arrowhead), and cerebellar tonsillar herniation (arrow).

**Figure 5:**
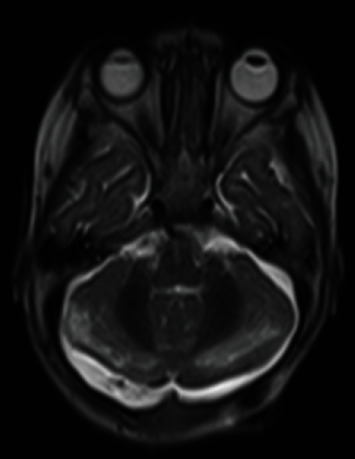
MRI evaluation 3 weeks after initial presentation. Axial T2-weighted image through the cerebellum three weeks after presentation shows volume loss and bright signal in the cerebellar hemispheres consistent with encephalomalacia in the regions of previous cerebellar injury and diffusion restriction.

Neurosurgery was consulted due to cerebellar swelling, and it was present in the PICU shortly after patient's arrival. The patient was started on a 3% hypertonic saline drip, and an EEG was obtained which showed diffuse background slowing with no seizure activity. The patient was closely monitored with serial neurologic exams. An MRI obtained two hours after presentation was concerning for persistent cerebellar swelling resulting in effacement of the fourth ventricle (Figure 3). The patient clinically worsened on hospital day 3, with increased somnolence, worsening ocular bobbing, intermittent bradycardia, and decreased movement of his upper extremities. An emergent head CT obtained on hospital day 3 showed increasing obstructive hydrocephalus (Figure 4). He was taken emergently to the operating room for decompressive suboccipital craniectomy with a patulous duroplasty and placement of an external ventricular drain (EVD). Postoperatively, he had a steadily improving neurological exam but the EVD was unable to be weaned. He was extubated 2 days after surgery and underwent ventriculoperitoneal shunt placement on hospital day 10, seven days after his initial surgery. He was discharged to acute rehabilitation and subsequently required a G-tube. A repeat MRI was obtained 3 weeks later (Figure 5) showing extensive encephalomalacia of the cerebellar hemispheres, in regions of previous cytotoxic injury.

On outpatient follow-up approximately 13 months later, the patient had normal motor strength and was walking although he remained very mildly apraxic and dysmetric. He was speaking well, majority of his calories are consumed by mouth with supplemental feeds via G-tube, and he was discharged from his physical, occupation, and speech therapies. He continues to follow with the developmental pediatric service who oversees his growth and developmental issues.

## 4. Discussion

This case highlights the existence of an uncommon yet severe presentation of opioid toxicity with acute cerebellar edema. Patients with this condition present with varying degrees of central nervous system depression, often including miosis, respiratory depression, lethargy, or obtundation, often with an associated acute neurological decline reported [7–9]. Initial imaging usually reveals cerebellar cytotoxic injury and swelling, which can be complicated by development of acute obstructive hydrocephalus and tonsillar herniation. This case as well as another from our institution caused by “Kommon” (tobacco leaves soaked in ketamine) joins a few other published cases in highlighting the existence of an uncommon yet severe syndrome of acute cerebellar cytotoxic edema in the setting of opioid or ketamine toxicity [10, 11]. Though there are few cases reported, a 2017 case series reported that four of 10 patients required posterior fossa craniectomy and CSF diversion with resultant good functional outcomes [11]. Both cases from our institution also underwent posterior fossa craniotomy and shunting with good functional outcomes. However, there is little information regarding pediatric patients with opioid overdose. In our case, cerebellar injury is likely a result of direct mu receptor-mediated cerebellar neurotoxicity rather than secondary to respiratory depression-induced hypoxic ischemic injury where the cerebellum is the last structure typically affected.

Mucosal application of transdermal fentanyl patches has resulted in significant morbidity and mortality since their development [12]. Intact ingested fentanyl patches can lead to longer duration of symptoms, as the gel reservoir contains a large amount of fentanyl that continues to be absorbed and the patches are often more difficult to remove from the body [9]. Furthermore, the mucosa has greater than 30-fold increase in fentanyl absorption due to its lack of stratum corneum that the skin provides, with rapid absorption of fentanyl into the blood stream [13–16]. Even after three days of typical transdermal use, the “used” fentanyl patch can still contain 28-84% of the original dose of fentanyl [17].

Unfortunately, timely diagnosis is difficult as there is often no provided history of opioid exposure and there is currently no definitive rapid testing available, so the clinician must have a high index of suspicion and perform a detailed physical examination. Radiological evaluation can assist in early diagnosis prior to toxicological results with CT findings of cerebellar edema, although the imaging findings may be confused with other more common causes of cerebellar edema such as ischemia or cerebellitis. Findings of obstructive hydrocephalus may be delayed [7, 18, 19]. As hydrocephalus can progress quickly in patients with limited neurologic exams, Reisner et al. recommend routine serial CT scanning for the first 48 hours for more timely identification of neuroradiographic sequelae [7]. While routine neurosurgical intervention is not recommended, if hydrocephalus is present, case outcomes show the importance of timely neurosurgical intervention [7, 10, 11, 20].

We present an uncommon yet severe syndrome of acute cerebellar cytotoxic edema in the setting of opioid or ketamine toxicity. Increased recognition of this condition by emergency room, neurosurgery, radiology, and critical care physicians is of paramount importance given the rising prevalence of use and abuse of these medications with the potential for children to access and ingest these medications. Patients need to be closely monitored for development of hydrocephalus and herniation syndromes, and timely surgical intervention appears to result in improved outcomes. A high index of suspicion for this diagnosis with early recognition and intervention has the potential to significantly improve patient's clinical outcomes.
